# Decreased Identification of Vesicoureteral Reflux: A Cautionary Tale

**DOI:** 10.3389/fped.2017.00175

**Published:** 2017-08-11

**Authors:** Aslam Hyder Qureshi, Oluwaseun Ajayi, Andrew Lawrence Schwaderer, David S. Hains

**Affiliations:** ^1^Innate Immunity Translational Research Center, Children Foundation Research Institute, Le Bonheur Children’s Hospital, Memphis, TN, United States; ^2^Department of Pediatrics, University of Tennessee Health Science Center, Memphis, TN, United States; ^3^Biomedical Informatics Core, Children’s Foundation Research Institute, Le Bonheur Children’s Hospital, Memphis, TN, United States; ^4^Division of Nephrology, Nationwide Children’s Hospital, Columbus, OH, United States

**Keywords:** vesicoureteral reflux, voiding cystourethrogram, renal scarring, pediatrics, renal bladder ultrasound

## Abstract

**Aim:**

To find the trend in patient’s visits to our centers for vesicoureteral reflux (VUR). We hypothesize that VUR diagnosis and hence possible nephropathy recognition may be diminishing because of changing practice patterns.

**Methods:**

Data were extracted from electronic medical records for new and follow-up patients aged 0–18 years with ICD-9/10 codes to correspond with VUR, VUR unilateral, VUR bilateral, and VUR with reflux nephropathy, as well as new patients with diagnoses of urinary tract infections (UTI) and pyelonephritis at two major pediatric centers from 2012 to 2015. Figures and statistics to reflect absolute clinic visits and annual trends were created with SPSS 2010. Linear regression was applied.

**Results:**

Annually, Le Bonheur Children’s Hospital and Nationwide Children’s Hospital experienced an average decrease of 13 and 17% in total VUR visits, and an average decrease of 22 and 27% in VUR nephropathy visits, respectively, for each institution. Patient visits for UTIs were reduced an average of 16% annually in both centers. Linear regression demonstrated that number of patients (patients/year ± SE) decreased annually 69 ± 19 (*P* = 0.02), 7 ± 2 (*P* = 0.02), and 67 ± 25 (*P* = 0.04) for VUR, VUR nephropathy, and UTI, respectively.

**Conclusion:**

We conclude that the decreased number of VUR and VUR nephropathy cases identified in subspecialty clinics (Nephrology/Urology) at two major children’s hospitals reflect a possible decreased identification of VUR. This trend may also be due to decreased referral of low grade cases of VUR. We cannot conclude that “undifferentiated UTI” referrals increased concomitantly to account for the decreased VUR as our data reflects a decreased trend in those visits as well. We suggest that clinicians following the American Academy of Pediatrics guidelines ensure that all UTI are accounted for and surveillance is appropriately escalated for recurrent UTI or abnormal imaging results.

## Introduction

Urinary tract infections (UTI) occur in 7% of children between 0 and 2 years of age (1). Imaging studies after UTI have shown vesicoureteral reflux (VUR) in 30–40% of children (2). In 1999, the American Academy of Pediatrics (AAP) guidelines recommended renal ultrasound and voiding cystourethrogram (VCUG) after first febrile UTI in children between 2 and 24 months of age to evaluate for urinary tract abnormalities. No recommendations were made on continuous antibiotic prophylactic (CAP) therapy (3). The AAP revised guidelines in 2011 recommend renal bladder ultrasound (RBUS) after first febrile UTI and VCUG after second febrile UTI or if RBUS is abnormal (4). Evidence from limited studies at the time did not indicate a role for CAP in preventing scarring (5). However, the risk of renal scarring increases exponentially from 10% with the second UTI to 60% following the sixth recurrent UTI (3). Recent evidence from Randomized Intervention for Children with Vesicoureteral Reflux (RIVUR) and Swedish VUR trials suggests definitive role of CAP in certain children with VUR in preventing recurrent UTIs (6, 7). In patients with VUR, recurrent UTIs are associated with acquired scarring risk (8, 9).

### Objective

We hypothesize that VUR diagnosis and hence possible nephropathy recognition may be diminishing because of changing UTI practice patterns. We collected data to determine the trends of UTI, VUR, and reflux nephropathy referral patterns. We analyzed trends in VUR diagnosis in subspecialty clinics (Nephrology/Urology) from 2012 to 2015 in two major pediatric tertiary care centers.

## Materials and Methods

Numbers of new and follow-up outpatient visits were extracted from electronic medical records for years 2012–2015 with primary and/or secondary diagnoses ICD-9/10 codes (593.70/N13.70, 593.71/N13.71, 593.72/N13.721/N13.722, and 593.73/N13.729) to correspond with VUR, VUR unilateral, VUR bilateral, and VUR with reflux nephropathy. Data were also collected for new visits for ICD-9/10 codes 590.10/N10 and 599.0/N39 to correspond to acute pyelonephritis and UTI, respectively. Data were collected from Le Bonheur Children’s Hospital (LBCH) in Memphis, TN, and Nationwide Children’s Hospital (NCH) in Columbus, OH. New cases were defined as first time visits for VUR diagnosis in either Urology or Nephrology clinics. Established cases were defined as those presenting for follow-up with previous visits in either clinic. Each patient was counted only once per year even if he/she had multiple follow-up visits that year. Data were collected for patients 0–18 years of age. Year-to-year and average percent change over the study period were calculated and graphs generated on IBM SPSS statistics 24. Linear regression analysis was done on SAS version 9.4.

## Results

**Figure 1:**
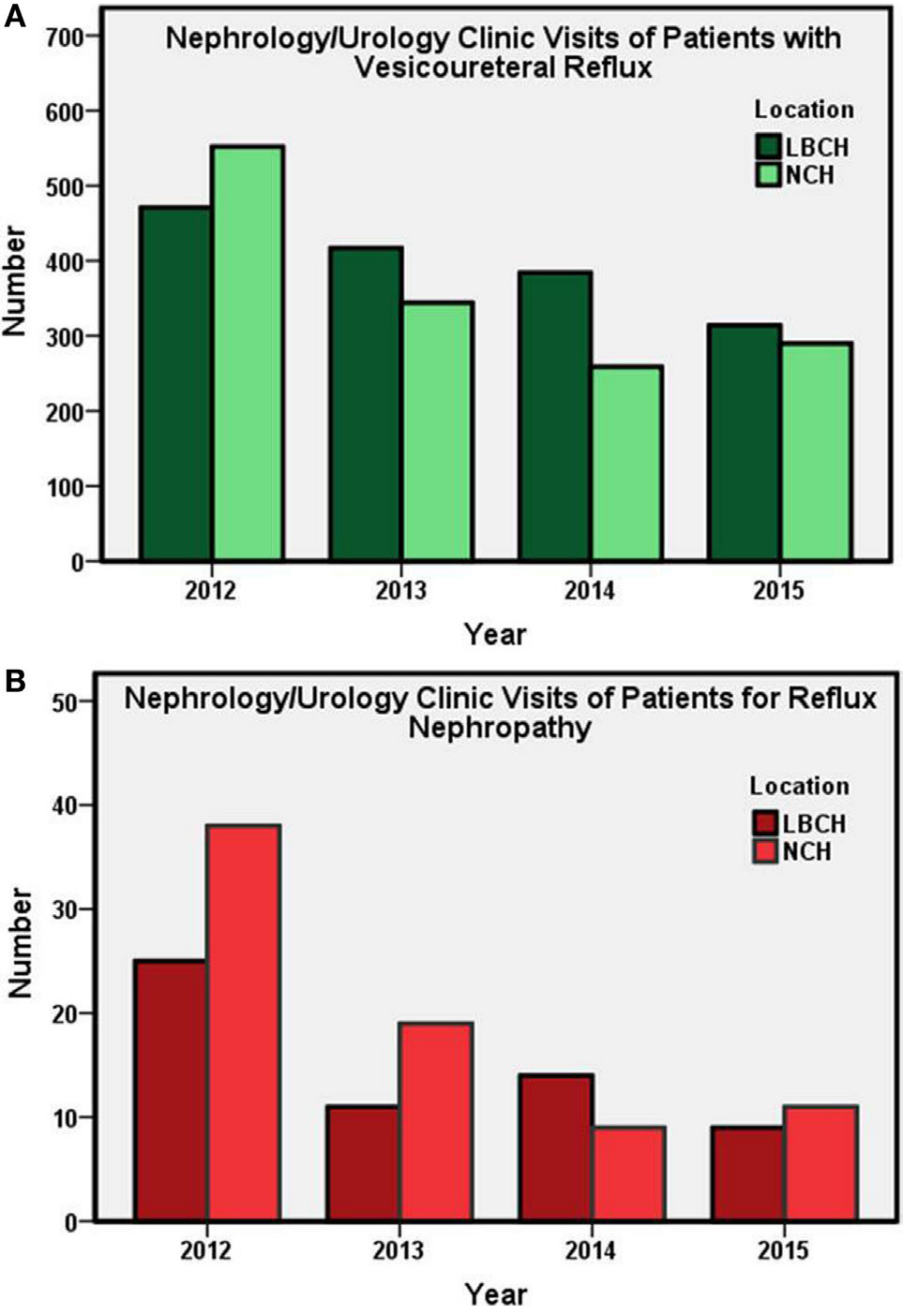
Subspecialty (Nephrology and Urology) clinic visits (total annual number) at LBCH and NCH from 2012 to 2015. **(A)** Annual subspecialty clinic visits of new and established patients with VUR. **(B)** Number of subspecialty clinic visits for patients with VUR nephropathy. LBCH, Le Bonheur Children’s Hospital; NCH, Nationwide Children’s Hospital.

**Figure 2:**
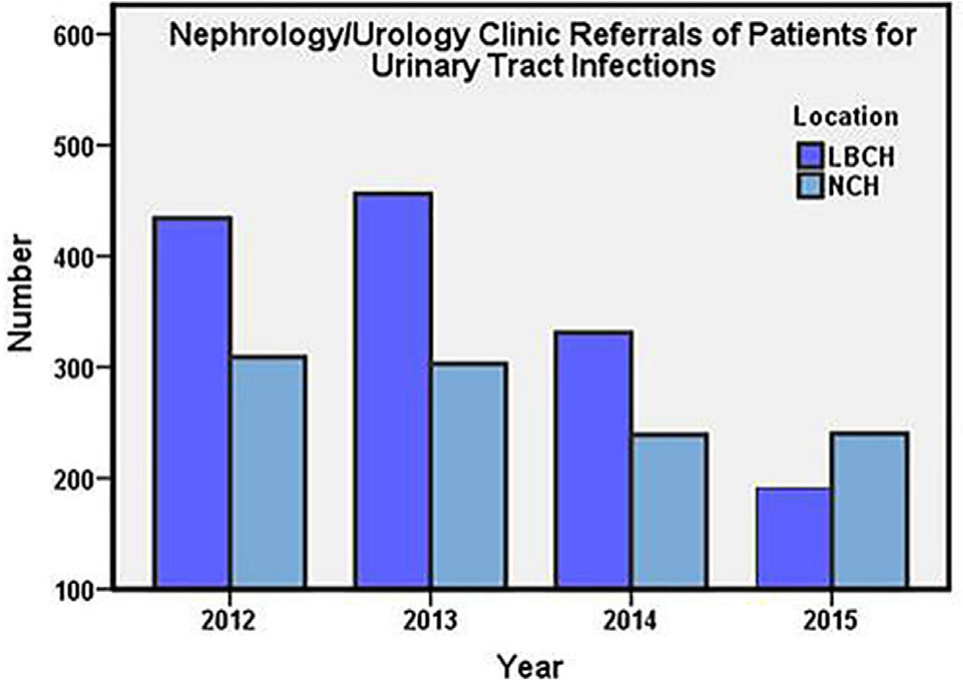
Subspecialty (Nephrology and Urology) clinic referrals (total annual number) of new patients at LBCH and NCH from 2012 to 2015. LBCH, Le Bonheur Children’s Hospital; NCH, Nationwide Children’s Hospital.

Annual numbers of patients visits for new and established cases of VUR and VUR nephropathy (Figure 1) and new patient referrals for UTI (Figure 2) from years 2012 to 2015 at both centers are presented. Annually, LBCH experienced an average 13% decrease in new and established cases during the study period. A comparable pediatric center from the Midwest, NCH, had an annual average decrease of 17% in new and established cases of VUR. From 2012 to 2015, LBCH and NCH experienced an average annual decrease of 22 and 27% in new and established VUR nephropathy cases, respectively. Visits for new cases of UTI averaged an annual decrease of 16% in both centers from 2012 to 2015. Linear regression demonstrated that number of patients (patients/year ± SE) went down 69 ± 19 (*P* = 0.02), 7 ± 2 (*P* = 0.02), and 67 ± 25 (*P* = 0.04) for VUR, VUR nephropathy, and UTI, respectively.

## Discussion

The AAP guidelines in 2011 were written with the objectives to diagnose and treat UTI in febrile children between 2 and 24 months of age and to initially screen for underlying renal anomalies by utilizing non-invasive diagnostic tests like RBUS avoiding more invasive diagnostic procedures like VCUG which require bladder catheterization and radiation exposure. The guidelines recommend using RBUS in children between 2 and 24 months of age after first febrile UTI and VCUG after the second febrile UTI. No specific guidelines exist for children older than 2 years of age. Our data suggest that the number of VCUGs performed following febrile UTIs may be decreasing leading to the decrease in VUR diagnoses. This reduced number of VCUGs may be a result of adherence to the 2011 AAP guidelines and supports other data recently published (10). Because, in our judgment, adherence to AAP guidelines would be optimized when children have close follow-up with a single primary care provider. Decreased VUR recognition may be concentrated in patients that are seen in multiple settings (Urgent care, Emergency department, and different primary care providers), thereby making application of the guidelines difficult. A third possibility is that VUR is increasingly being managed in the primary care setting, which is also supported by our finding of decreasing trend in the UTI visits at both centers. After 5 years since these guidelines were released, no new evidence has emerged indicating an ideal treatment to prevent renal scarring in patients with VUR although there is some evidence for use of CAP in preventing recurrent UTIs (6).

Our data show reduction in the cases of VUR nephropathy. From a nephrologist’s point of view, an unwanted consequence of decreased VUR surveillance would also be decreased surveillance of sequelae such as renal scarring. We have identified several potential explanations for this aforementioned trend including (1) decreased diagnosis of VUR and/or VUR nephropathy due to decreased surveillance, (2) misinterpretation or incorrect application of VUR guidelines resulting in less VUR nephropathy diagnosis and management, (3) decrease in VUR nephropathy occurrence in the general population, which is very unlikely, and/or (4) increased management of VUR nephropathy in the primary care setting, which could be in view of previous studies showing no evidence of medical or surgical management changing the progression of VUR nephropathy and even questioning the role of VUR in nephropathy (11, 12). On the other hand, evidence does suggest that the number of UTIs is associated with increased risk of scarring especially in girls with VUR (3, 13).

In order to diagnose reflux nephropathy, a patient must have radiologic evidence of parenchymal abnormalities consistent with renal scarring. Additionally, new onset hypertension and/or CKD in the context of VUR would suggest reflux nephropathy and could result in ICD-9/10 coding, accordingly. We acknowledge that the lack of availability of DMSA may have resulted in decreased recognition, but our centers have employed additional imaging techniques to monitor/diagnose renal parenchymal abnormalities when clinically appropriate. While we cannot specifically analyze each case presented for the rationale for giving a diagnosis of reflux nephropathy, we cannot attribute our trends to changing clinical criteria for diagnosing VUR nephropathy. The downward trend in VUR nephropathy diagnoses is the most concerning finding in our study. While we acknowledge that this data is based on ICD-9/10 coding and has inherent limitations, we cannot exclude the possibility that the trend is real. Furthermore, treatment strategies have not changed over the study period to explain a true decrease in VUR nephropathy. However, RBUS has a relatively low sensitivity for VUR and VUR nephropathy, and results of RBUS are also operator and patient dependent. A recent study showed the sensitivity and negative predictive values of RBUS for Grades I–V VUR were 52.3 and 75.1%, and for Grades III–V VUR were 68.4 and 87.8%, respectively (14). Two studies showed poor correlation between RBUS and VCUG (15, 16). RBUS also has poor sensitivity to detect renal scars (17). While influenced by grade of VUR, scarring risk is not isolated to only high-grade VUR (18). Thus, ignoring low-grade VUR could lead to missed VUR nephropathy recognition and support the trends we present. Our data show an upward trend in the 2015 cases that could have resulted from the transition from ICD-9 to ICD-10 coding. ICD-10-CM provides better capture of diagnoses that are missed by ICD-9-CM, and extensive training and courses on ICD-10-CM may have had effect on reporting more efficiently in 2015 (19).

### Limitations

These study data are based on ICD-9/10 coding and have inherent limitations that depend upon correct code entry in the system. This small study is based upon findings of two large centers and trends at other institutions in North America are needed.

## Conclusion

While the clinical approach to VUR has not changed to result in actual decreased incidence of disease, we conclude that the decreased number of VUR and VUR nephropathy cases identified in subspecialty clinics at two major children’s hospital reflect a decreased referral for UTI and VUR to specialty clinics by primary care physicians. Based on AAP guidelines, the drop in the diagnosis of VUR may reflect reduced identification of minor cases that do not have long-term ramifications for kidney health. On the other hand, the data may reflect missing cases of VUR and nephropathy in the community that will be seen in future in trends in the causes of pediatric end-stage renal disease. We suggest that clinicians following the AAP guidelines ensure that all UTI are accounted for and surveillance is appropriately escalated for recurrent UTI or abnormal imaging results. We recommend counseling parents about the symptoms of UTI such as fever, dysuria, red color urine, and abdominal or flank pain and advising parents to seek medical advice in that situation. Moreover, if parents seek medical care for their child outside of the medical home, they should update the primary care physician about any new episodes of UTI. Thus, clinical surveillance can be appropriately escalated and appropriate imaging obtained and/or subspecialty referrals can be made. Given that we only present data from two medical centers, and factors like grade of VUR and evidence of nephropathy could not be specified in each case due to the nature of study, future prospective, multicenter studies are needed to investigate these trends.

## Ethics Statement

This study is from de-identified data pulled from electronic medical record database. No IRB/informed consent was necessary for data collection.

## Author Contributions

AQ, AS, and DH provided the concept and structure for the study. All authors involved in data analysis and interpretation. OA extracted the data. AS prepared the first draft of manuscript. AS and DH reviewed and revised the manuscript. All authors approved the final version of manuscript.

## Conflict of Interest Statement

AS has consulted for Allena Pharmaceuticals. All other authors have no conflict of interest.
